# Effectiveness of a Commercial Lactic Acid Bacteria Intervention Applied to Inhibit Shiga Toxin-Producing* Escherichia coli* on Refrigerated Vacuum-Aged Beef

**DOI:** 10.1155/2017/8070515

**Published:** 2017-05-23

**Authors:** Katie R. Kirsch, Tamra N. Tolen, Jessica C. Hudson, Alejandro Castillo, Davey Griffin, T. Matthew Taylor

**Affiliations:** ^1^Department of Nutrition and Food Science, Texas A&M University, College Station, TX, USA; ^2^Department of Animal Science, Texas A&M University, College Station, TX, USA; ^3^Department of Animal Science, Texas A&M AgriLife Extension, College Station, TX, USA

## Abstract

Because of their antagonistic activity towards pathogenic and spoilage bacteria, some members of the lactic acid bacteria (LAB) have been evaluated for use as food biopreservatives. The objectives of this study were to assess the antimicrobial utility of a commercial LAB intervention against O157 and non-O157 Shiga-toxigenic* E. coli* (STEC) on intact beef strip loins during refrigerated vacuum aging and determine intervention efficacy as a function of mode of intervention application. Prerigor strip loins were inoculated with a cocktail (8.9 ± 0.1 log_10_ CFU/ml) of rifampicin-resistant (100.0 *μ*g/ml; Rif^R^) O157 and non-O157 STEC. Inoculated loins were chilled to ≤4°C and treated with 8.7 ± 0.1 log_10_ CFU/ml LAB intervention using either a pressurized tank air sprayer (conventional application) or air-assisted electrostatic sprayer (ESS). Surviving STEC were enumerated on tryptic soy agar supplemented with 100.0 *μ*g/ml rifampicin (TSAR) to determine STEC inhibition as a function of intervention application method (conventional, ESS) and refrigerated aging period (14, 28 days). Intervention application reduced STEC by 0.4 log_10_ CFU/cm^2^ (*p* < 0.05), although application method did not impact STEC reductions (*p* > 0.05). Data indicate that the LAB biopreservative may assist beef safety protection when utilized within a multi-intervention beef harvest, fabrication, and aging process.

## 1. Introduction

An estimated 175,905 Shiga-toxigenic* Escherichia coli* (STEC) foodborne disease cases occur in the United States each year, with non-O157 STEC being reportedly the causative agents in 64.1% of cases [1]. The US Department of Agriculture Food Safety and Inspection Service (USDA-FSIS) declared raw nonintact beef, as well as intact beef intended to be processed into nonintact beef, adulterated if found positive for* E. coli* belonging to serogroups O26, O45, O103, O111, O121, O145, and/or O157 [2]. Cattle serve as a reservoir of STEC [3–5]; eradication of these pathogens from the beef supply chain remains a challenge. Chemical food safety interventions such as lactic acid, peroxyacetic acid, and chlorine are commonly applied to reduce and/or eliminate spoilage and pathogenic organisms from beef surfaces [6–8]. However, consumer demand for natural or minimally processed foods [9, 10] suggests a need for alternative beef safety interventions [11].

The lactic acid bacteria (LAB), as a type of biopreservative, are reported to be useful for preventing the growth of pathogenic microbes on meat products [12, 13] and are in some instances classified as generally recognized as safe (GRAS) for use in nonintact, whole muscle cuts or carcasses, and ready-to-eat meats [14, 15]. These organisms antagonize other bacteria, including human pathogens, through competition for nutrients and/or attachment sites, production of antimicrobial metabolites (e.g., reuterin, diacetyl, and fatty acids), bacteriocins (e.g., nisin, pediocin), and weak organic acids (e.g., lactic, acetic acid) [16, 17]. Previous studies have explored the inhibitory mechanisms of specific protective cultures from members of the LAB for inhibition of* E. coli *O157:H7 in meats [13, 18–25]. Nevertheless, to date little data are published detailing the antimicrobial efficacy of LAB food safety interventions for inhibiting members of the non-O157 STEC on fresh beef during storage and handling prior to retail. Therefore, the objectives of this study were to (i) assess the efficacy of a commercial LAB biopreservative (LactiGuard™, Guardian Food Technologies, LLC, Overland Park, KS) for the inhibition of eight STEC serogroups on beef subprimals during refrigerated vacuum aging and (ii) determine whether mode of intervention application (conventional spray, electrostatic spray [ESS]) impacts antimicrobial efficacy.

## 2. Materials and Methods

### 2.1. Bacterial Culture Maintenance

Rifampicin-resistant (Rif^R^) STEC strains encompassing O157 and the six O-groups of non-O157 STEC named as adulterants in raw nonintact beef [2], and* E. coli *O104:H4 (2011 European sprout outbreak) (STEC8), were provided by J. B. Luchansky, Ph.D. (US Department of Agriculture-Agricultural Research Service, Wyndmoor, PA, USA) (Table 1). Culture revival and maintenance procedures were completed according to previous methods [26]. Working cultures of isolates were prepared by transferring a loopful of culture from tryptic soy agar (TSA; Becton, Dickinson and Co., Sparks, MD, USA) slants into 10 ml sterile tryptic soy broth (TSB) and incubating statically at 35°C for 18–24 h. Each isolate was individually subcultured by inoculating a 50 ml volume of sterile TSB supplemented with 0.1% (w/v) rifampicin (Sigma-Aldrich, St. Louis, MO, USA) with one loopful of fresh culture and incubating statically at 35°C for 18–24 h. Immediately prior to use, a cocktail of STEC isolates was prepared by transferring 50 ml of each culture into a calibrated misting bottle (previously sanitized by immersion in 70% ethanol for 5 min followed by triplicate flushing with sterile distilled water). The targeted STEC concentration in the inoculum was 9.0 log_10_ CFU/ml and was determined by serially diluting in 0.1% peptone water (Becton, Dickinson and Co.) and spreading on TSA supplemented with 100 *μ*g/ml rifampicin (TSAR). Colonies were enumerated following incubation at 35°C for 18–24 h. Inoculum preparation procedures were completed to provide nondiffering counts of each of the eight* E. coli *isolates by mixing equivalent volumes into the inoculation bottle and thoroughly mixing isolates together prior to application. Isolates were prepared for inoculation according to methods previously published by Kirsch et al. [26], who reported that STEC isolates identical to those used in the current study were able to achieve non-statistically differing counts following 24 h incubation at 35°C in a nutritious medium. Rif^R^ organisms used in the current study were previously compared to antibiotic-sensitive parents for tolerance to common food safety interventions including heating, lactic acid exposure, and high pressure, with no statistically significant differences in survivors between parents and mutants [27].

The commercial biopreservative LactiGuard consisted of lyophilized powders containing a manufacturer-described set of* Lactobacillus*,* Lactococcus*, and* Pediococcus* spp. Immediately prior to use, a LactiGuard suspension was prepared by combining 7.5 g (11.0 log_10_ CFU/g) of each organism (4 total) into 3.0 liters of sterile water to a concentration of 8.4 log_10_ CFU/ml. LAB numbers in the antimicrobial suspension prior to application to beef surfaces were verified by enumerating on de Man, Rogosa, and Sharpe (MRS) lactobacilli agar (Becton, Dickinson and Co.) supplemented with streptomycin sulfate (40 *μ*g/ml, Amresco, Solon, OH, USA), sodium oxacillin (0.4 *μ*g/ml, Chem-Impex Int., Inc., Wood Dale, IL, USA), and gentamycin sulfate (5 *μ*g/ml, Amresco), as per intervention supplier instructions. Colonies were enumerated following anaerobic incubation for 48 h at 35°C.

### 2.2. Meat Preparation and Inoculation

Prerigor beef strip loins were procured from a federally inspected establishment in Texas and harvested within 2 h of animal death. Once collected, each strip loin was transferred into a polyethylene bag and swathed in a thermal blanket (EverReady First Aid, Brooklyn, NY, USA) to minimize heat loss. Beef pieces were then immediately transported in insulated coolers containing activated instant hot packs (Dynarex, Orangeburg, NY, USA) to a Texas A&M AgriLife Research facility maintaining a BSL-2 Laboratory located within a 30 min drive of the beef slaughter establishment to inoculate strip loins as quickly as possible after collection (simulating prerigor carcass cross-contamination during beef harvest). At the facility, a prepared inoculum application spray bottle was primed and held 25 to 31 cm above the meat for application of three pumps of inoculum (1.0–1.5 ml per pump) onto the lean side of the strip loin surface. The bag containing the inoculated strip loin was closed with a zip tie and hand-tumbled for 1 min to distribute inoculum over beef surfaces. Beef pieces were held at 25°C for 30 min for inoculum attachment, loaded into a second polypropylene bag, and placed in insulated coolers containing frozen ice packs to initiate chilling. Coolers were transported to the Food Microbiology Laboratory (Texas A&M University) within 8 h of inoculation and bacterial attachment. Upon return, bagged strip loins were removed from coolers and placed on shelves in a single layer in a 4°C walk-in cooler and held until a total chilling period of 24 h had elapsed.

### 2.3. Intervention Application to Beef

Following chilling, inoculated beef strip loins were readied for intervention application (under biosafety level 2 [BSL2] containment) and sample analysis. Prior to treatment application, a sterile meat hook was inserted into the distal end of the strip loin and strip loins were hung lean side facing outward in a model spray cabinet (Birko Corp., Centennial, CO, USA). Each piece was randomly assigned to a treatment: (a) conventionally spray-applied LactiGuard [25°C for 100 s at 310 kPa, 1.7 ml/min flow rate]; (b) ESS-applied LactiGuard [25°C for 120 s at 207 kPa]; or (c) STEC-inoculated, untreated control. ESS application was performed using a XT-3 air-assisted ESS sprayer (Electrostatic Spraying Solutions, Inc., Watkinsville, GA, USA) charged to ≤−10 amps at a flow rate of 2.1 ml/s, while the conventional spray application was achieved using a hand-held, pressurized tank air sprayer (Roundup, Marysville, OH, USA) at a flow rate of 1.7 ml/s. Interventions were sprayed approximately 90 cm from the strip loin surface in a sweeping horizontal zig-zag motion. Strip loins were then inserted into commercial-grade vacuum bags (oxygen transmission rate: ≤50 cm^3^/m^2^·24 h·0.1 MPa; Weston, Strongville, OH, USA) and packaged in a vacuum sealer. Strip loins were arranged in a single layer and stored at 4°C for 14 or 28 days prior to postaging sampling.

### 2.4. Sampling and Microbiological Analysis

In order to track changes in meat pH as a function of intervention application, external pH of individual strip loins was measured in triplicate using an ExStik® pH and temperature meter (Extech Instruments Corp., Nashua, NH, USA) before and after STEC inoculation, before and after LAB intervention treatment, and again after 14 and 28 days of refrigerated vacuum aging. For microbiological sampling, three 10 cm^2^ outlines were marked on the lean tissue surface using a flame-sterilized stainless steel borer, excised to a depth of 1-2 mm using flame-sterilized scalpels and forceps, and composited into a sterile stomacher bag. Samples were then sealed and transported in insulated coolers packed with ice to the Food Microbiology Laboratory for analysis. Beef samples were assayed by adding 99 ml phosphate buffered saline (PBS; Sigma-Aldrich Co.) to each sample pouch, pummeling for 1 min in a stomacher, serially diluting in 0.1% peptone water, and spreading on an appropriate medium. LactiGuard LAB were enumerated on MRS agar supplemented with antibiotics described above and incubated anaerobically 48 h at 35°C prior to colony counting. Rif^R^ STEC were spread on TSAR and colonies enumerated following incubation at 35°C for 24 h.

### 2.5. Statistical Analysis

The experiment was completed via triplicate identical samples conducted over two replications (*N* = 6). All statistical analyses were performed using JMP Pro v11.0 (SAS Institute, Inc., Cary, NC, USA). Colony counts were transformed to log_10_ CFU/cm^2^; the limit of detection for the plating assays was 0.5 log_10_ CFU/cm^2^. Differences between main effects (vacuum aging duration, and biopreservative intervention application method [control, conventional spray, ESS]) and their interaction were identified by analysis of variance (ANOVA) at a *p* = 0.05. Statistical differences among means were separated with Tukey's Honestly Significant Differences (HSD) multiple comparisons test (*p* < 0.05).

## 3. Results and Discussion

To simulate cross-contamination during commercial animal slaughter prior to intervention application, beef strip loins were collected within 2 h of animal slaughter and inoculated as rapidly as possible. STEC inoculum fluid contained 8.9 ± 0.1 log_10_ CFU/ml prior to application to beef surfaces; STEC populations on inoculated strip loins remained unchanged during chilled transportation to the food microbiology laboratory prior to treatment (Table 2). Although control strip loins (STEC-inoculated, untreated) were handled identically to those subjected to LAB intervention treatment, STEC numbers enumerated from nontreated controls after chilling (6.6 ± 0.1 log_10_ CFU/cm^2^) were statistically lower than those from intervention-treated samples (7.2 ± 0.1 log_10_ CFU/cm^2^) (*p* < 0.05) (Table 2). This was unexpected; authors are uncertain as to the cause(s) behind this observed difference in STEC counts between controls and other samples eventually treated with LAB.

Table 2 presents surviving populations of STEC and LAB on strip loins immediately following treatment during vacuum aging at 4°C. Analysis of microbiological data determined method of intervention application (conventional spray, ESS) did not influence LAB numbers recovered from treated beef strip loins (6.5 ± 0.1 log_10_ CFU/cm^2^). Likewise, STEC numbers on treated strip loins did not differ as a function of the mode of LAB application method (*p* ≥ 0.05). Nevertheless, STEC numbers on biopreservative-treated strip loins differed by intervention application with respect to STEC counts before and after treatment on strip loins and by posttreatment aging period (*p* = 0.015) (Table 2). Applying LAB onto beef strip loins resulted in a reduction in STEC of 0.4 ± 0.1 log_10_ CFU/cm^2^ (*p* < 0.05). Once treated, subprimals were individually vacuum packaged and aged at 4°C for 14 or 28 days. On day 14 posttreatment, STEC numbers increased to 7.1 log_10_ CFU/cm^2^ and were not different from STEC counts obtained immediately after intervention applications. Conversely, at 28 days of vacuum refrigerated aging, STEC counts (6.7 ± 0.1 log_10_ CFU/cm^2^) were significantly lower than pretreatment means (*p* < 0.05). At 14 days' vacuum refrigerated aging, LAB numbers on treated strip loin surfaces declined from posttreatment application counts to 6.2 log_10_ CFU/cm^2^, though no further changes in LAB counts were detected at day 28 (*p* ≥ 0.05) (Table 2). Overall, reductions in STEC counts following intervention application and refrigerated aging were modest and not likely of great antimicrobial significance.

Outcomes from previous studies evaluating the antimicrobial activity of LAB biopreservatives against pathogens on beef products are mixed with respect to pathogen reduction results. Smith et al. [22] inoculated ground beef with 5.0 log_10_ CFU/g of* E. coli *O157:H7 and 7.0 log_10_ CFU/g of LAB (*Lactobacillus acidophilus* strains NP 51, NP 25, NP 7, and NP 3) and then stored the product at 5°C under vacuum for up to 5 days prior to analysis of pathogen survival. Compared to untreated controls,* E. coli* O157:H7 in treated beef was reduced by >3.0 log_10_ CFU/g following refrigeration for 5 days (*p* < 0.05). Echeverry et al. [28], conversely, reported that* E. coli *O157:H7 inoculated on refrigerated strip loins (5.0 log_10_ CFU/cm^2^) treated with a spray consisting of 7.7 log_10_ CFU/ml LAB remained unchanged during refrigerated vacuum aging over 21 days, similar to findings reported herein. Finally, previous research has reported that reductions in* E. coli* O157:H7 on beef following treatment with LAB intervention were independent of LAB numbers applied, with no differences in* E. coli* O157:H7 numbers observed following incubation [29]. In the current study, numbers of LAB and STEC were nearly equivalent throughout the refrigerated aging period, yet LAB did not exert strong pathogen inhibition activity (Table 2). This may have resulted from insufficient levels of fermentable sugars for production of organic acids, lack of available oxygen for production of peroxides, or possibly other unanticipated factors. Finally, no effort to differentially count surviving STEC from intervention-treated or control strip loins during the experiment was attempted, disallowing authors from determining whether some members of the STEC inoculum were inhibited to greater or lesser extents than other inoculum members. While Kirsch et al. [26] reported differing morphological characteristics of STEC strains identical to those used herein on selective/differential media, it was noted that STEC isolates not bearing good isolation were frequently subject to misidentification on selective/differential plating medium surfaces. The enumeration of total surviving STEC from the inoculum would be expected to primarily yield counts of STEC isolates most tolerant to the antimicrobial intervention, though quantification of any differences in inhibition on individual STEC isolates was therefore precluded.

Mean surface pH of strip loins immediately prior to inoculation was 6.2 ± 0.1, while pH following meat inoculation and chilling of inoculated product declined to 5.8 ± 0.1 (*p* < 0.05) (data not shown). Whereas application of the LAB intervention by conventional pressurized sprayer versus ESS did not produce statistically significant differences in meat surface pH, surface pH significantly decreased from the point of treatment application (LAB, control) to 14 days of refrigerated vacuum storage (5.4 ± 0.1) (*p* < 0.05). A small, nonstatistically significant decline in meat surface pH was observed to occur between days 14 and 28 for refrigerated vacuum-aged strip loins (5.3 ± 0.1) (*p* > 0.05) (data not shown). These pH declines mirror those for similarly chilled and vacuum-aged refrigerated beef in other studies where researchers determined LAB-driven declines in beef meat pH during chilled aging [30–32]. The inhibition of STEC by LAB is thought to result largely from production of organic acids [33]. However, exposure to sublethal stress (cold storage, carbohydrate limitation) may have limited acid fermentation output of LAB [34, 35]. Data indicate that acid fermentation of endogenous carbohydrate by applied LAB was insufficient to exert a greater degree of pathogen inhibition than that observed. This study investigated the efficacy of a commercial LAB food safety biopreservative intervention for inhibiting growth of eight O157 and non-O157 STEC isolates on beef strip loins during refrigerated vacuum aging. STEC numbers were reduced following treatment, and STEC populations on beef strip loins after 28 days of aging were lower than at pretreatment. However, pathogen reductions were small (<1.0 log_10_-cycle). While not likely effective as a sole antimicrobial intervention for beef safety protection, biopreservative food safety interventions such as LactiGuard can be utilized to gain useful reductions in STEC when integrated into a multi-intervention process for beef safety protection during beef harvest and fresh beef products manufacture.

## Figures and Tables

**Table 1:** Shiga-toxigenic *Escherichia coli *isolate identification and sources.

STEC serotype	Isolate ID^a^	Source
O104:H4	TY-2482 ATCC BAA-178	Human stool
O157:H7	USDA-FSIS 380-94	Salami isolate
O26:H11	H30	Infant with diarrhea
O103:H2	CDC 90-3128	Human stool
O45:H2	CDC 96-3285	Human stool
O145:NM	83-75	Human stool
O111:H-	JB1-95	Clinical isolate
O121:H19	CDC 97-3068	Human stool

^a^Isolates were provided by Luchansky, Ph.D. (USDA-Agricultural Research Service, Wyndmoor, PA).

**Table 2:** Least squares means of bacterial populations on beef strip loins (log_10_ CFU/cm^2^) treated with lab and vacuum aged at 4°C.

Target organisms	Experimental process stage^a^					*p* value
	Postinoculation	Postchilling	Posttreatment	14 days of aging	28 days of aging	
Control^b^	6.7A	6.6A	—	6.6A	6.3A	0.4047
STEC^c^	—	7.2A	6.8B	7.1AB	6.7B	0.0151
Lactic acid bacteria (LAB^d^)	—	—	6.5A	6.2B	6.1B	0.0124

^a^Values are least square means from two replications with triplicate samples in each replication (*n* = 6). Means within a row lacking the same capitalized letter (A, B) differ at *p* = 0.05 by Tukey's Honestly Significant Differences (HSD) multiple comparisons test. ^b^Control indicates STEC counts from STEC-inoculated, nontreated beef strip loins. STEC were enumerated on tryptic soy agar supplemented with 100.0 *μ*g/ml (TSAR) following 48 h incubation at 35°C. ^c^STEC denotes STEC means from strip loins treated with the LAB intervention by pressurized spray or ESS. STEC were enumerated on tryptic soy agar supplemented with 100.0 *μ*g/ml (TSAR) following 48 h incubation at 35°C. Significant differences in STEC counts were not detected as a function of intervention application by pressurized spray versus ESS; counts of organisms are therefore compiled for both application methods; ^d^LAB denotes numbers of LAB enumerated from intervention-treated strip loins (pressurized spray, ESS). As significant differences in LAB counts were not detected as a function of intervention application by pressurized spray versus ESS, counts of organisms were compiled for both application methods. LAB from the biopreservative LactiGuard LAB were enumerated on de Man, Rogosa, and Sharpe (MRS) agar supplemented with streptomycin sulfate (40 *μ*g/ml), sodium oxacillin (0.4 *μ*g/ml), and gentamycin sulfate (5 *μ*g/ml), as per manufacturer guidance.
